# Nanozyme Applications: A Glimpse of Insight in Food Safety

**DOI:** 10.3389/fbioe.2021.727886

**Published:** 2021-08-24

**Authors:** Long Wu, Shuhong Zhou, Gonglei Wang, Yonghuan Yun, Guozhen Liu, Weimin Zhang

**Affiliations:** ^1^ College of Food Science and Engineering, Hainan University, Haikou, China; ^2^ Key Laboratory of Fermentation Engineering (Ministry of Education), College of Bioengineering and Food, Hubei University of Technology, Wuhan, China; ^3^ School of Life and Health Sciences, The Chinese University of Hong Kong, Shenzhen, China

**Keywords:** Food safety, artificial enzyme, colorimetric assays, peroxidase activity, enzyme mimics

## Abstract

Nanozymes own striking merits, including high enzyme-mimicking activity, good stability, and low cost. Due to the powerful and distinguished functions, nanozymes exhibit widespread applications in the field of biosensing and immunoassay, attracting researchers in various fields to design and engineer nanozymes. Recently, nanozymes have been innovatively used to bridge nanotechnology with analytical techniques to achieve the high sensitivity, specificity, and reproducibility. However, the applications of nanozymes in food applications are seldom reviewed. In this review, we summarize several typical nanozymes and provide a comprehensive description of the history, principles, designs, and applications of nanozyme-based analytical techniques in food contaminants detection. Based on engineering and modification of nanozymes, the food contaminants are classified and then discussed in detail via discriminating the roles of nanozymes in various analytical methods, including fluorescence, colorimetric and electrochemical assay, surface-enhanced Raman scattering, magnetic relaxing sensing, and electrochemiluminescence. Further, representative examples of nanozymes-based methods are highlighted for contaminants analysis and inhibition. Finally, the current challenges and prospects of nanozymes are discussed.

## Introduction

Nanomaterials have received widespread attention in fields like chemistry, biology, environment, medicine and health, aerospace, resources and energy, and so on (Huang et al., 2019d; Wu et al., 2019a; Sun et al., 2018). Regarding their specific properties on mechanical, electrical, magnetic, optical, and catalytic activities, all kind of nanomaterials have been prepared and studied. Nanozyme, a kind of specific nanomaterials with enzyme-mimicking activity, is more and more favored by researchers. Nanozymes have shown a broad range of applications *in vitro* detection and living systems (Liang and Yan 2019). They hold a promise to serve as direct surrogates of natural enzymes in the analytical methods, especially the immunoassays (Wu et al., 2019b).

Compared with natural enzymes, nanozymes are easier to be modified and purified. Besides, the size, morphology (e.g., nanospheres, nanosheet, nanorods, nanowires, etc.), and surface groups can contribute to the enzyme-like activity of nanozymes (Wang et al., 2019; Gao et al., 2020). In this regard, nanozymes could thus be flexibly used as an effective medium in the construction of analytical methods. As a particular kind of nanomaterials, nanozymes are usually conjugated with antibody or DNA sequences to construct signal amplification strategy (Zhu et al., 2017; Tao et al., 2020). When combined with traditional concepts of optical, electrochemical, or colorimetric assays, the analytical methods with lower detection limits can be developed.

On the other hand, food contaminants, a kind of toxic substances that is harmful to human, have increasingly grown in complexity and followed up on new public health issues, novel safety emergencies, and emerging consumer demands (Huang et al., 2019a). The complexity of the pollutants and food matrices brings great challenge to the analytical methods. For instance, *Alternaria* can generate several toxic secondary metabolites, like alternariol, alternariol monomethyl ether, altenuene, tentoxin, and tenuazonic acid, which are widely found in sorghum, sunflower seeds, cereals, tomatoes, wine, beers, apple juices, and beverages (Pinto and Patriarca 2017). Various analytical strategies have been developed to monitor their occurrence in foods or food production chain. Obviously, those wet-chemistry-based analytical methods have been gradually replaced by powerful techniques that enable high enhancements in accuracy, precision, and detection limits. The new technologies can get over difficulties of conventional methods, such as time-consuming analysis, laborious procedures, and high cost.

**FIGURE 1:**
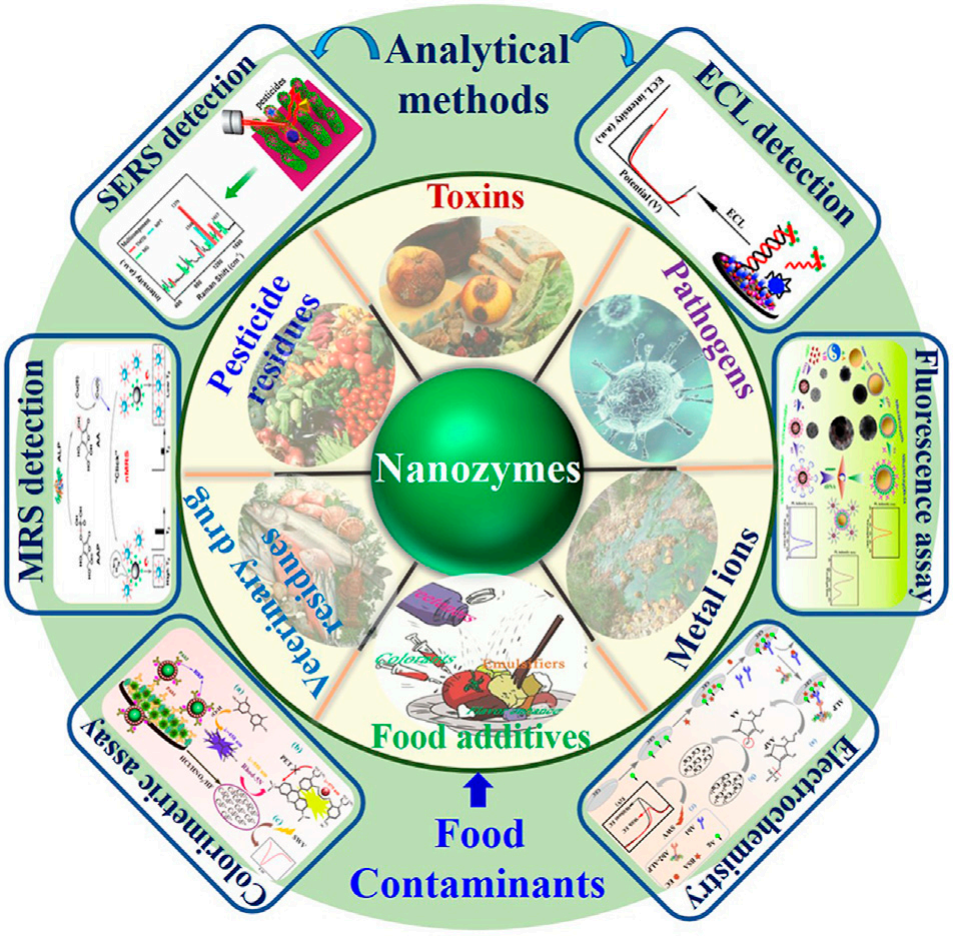
The schematic presentation of nanozymes-based analytical techniques for the detection of different food contaminants (MRS, magnetic relaxing sensing; SERS, surface enhanced Raman scattering; ECL, electrochemiluminescence).

The development of novel “rapid” detection methods has decreased detection time dramatically and thus could solve the main concerns of most of the analytical methods. Hence, a new frontier of nanozymes in food contaminants detection gives a glimpse of insight in this concept (Figure 1). In this review, an overview of emerging methods based on nanozymes is provided, with a focus on their varieties, surface modifications, and applications in food contaminants analysis.

## History and Development

**FIGURE 2:**
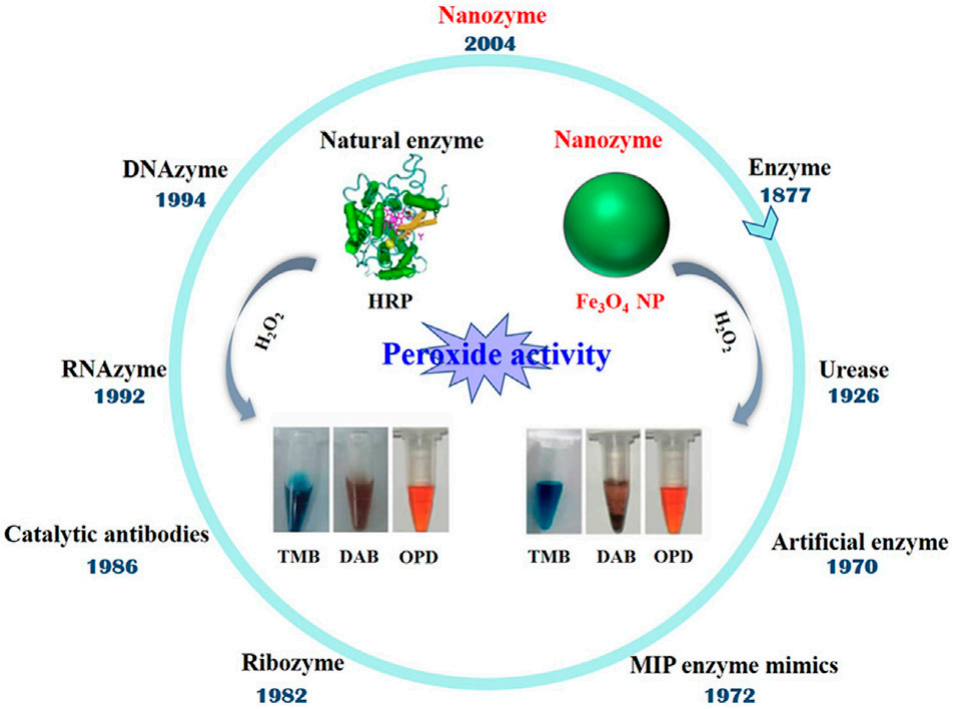
The history of nanozyme in enzyme mimics and the evidence of Fe_3_O_4_ NP behaving peroxide activity (inset).

Looking back at the first term “enzyme” coined by Wilhelm Kuhne in 1877, enzymes have gone for more than 120 years. Manea et al. described the transphosphorylation reactivity of triazacyclononane-functionalized Au NP, and the new term “nanozyme” was coined (Manea et al., 2004). Since then, the nanozymes have become a new member in field of enzyme mimics. Later, Gao et al. discovered that Fe_3_O_4_ NP has good enzyme-mimicking activity, which opens up a broad range of applications (Gao et al., 2007). Now, it is known that nanozymes are nanomaterials with intrinsic enzyme-like characteristics. The development and evidence of nanozymes behaving peroxide activity are shown in Figure 2. Given advantages like low cost, recyclable utilization, high catalytic activity, and stability, nanozymes are expected to be the next-generation artificial enzymes (Ⅱ).

**FIGURE 3:**
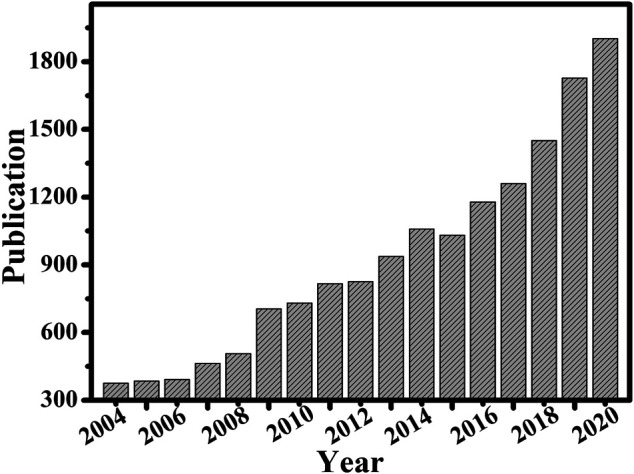
Number of published papers on nanozyme research by the end of 2020. The data is based on the Web of Science.

In recent decades, more than 200 research groups around the world have been studying nanozymes (Liang and Yan 2019). However, the activity descriptors of the nanozymes still remain largely unknown. Enormous efforts have been devoted to exploring nanozymes covering hundreds of nanomaterials. Dramatic growth has been witnessed for nanozymes research in catalysis, analytical techniques, environmental science, biomedical diagnosis, bioimaging, and antibacterial agents, suggesting their scientific significance and application prospects (Figure 3).

## Classification

### Classification

**FIGURE 4:**
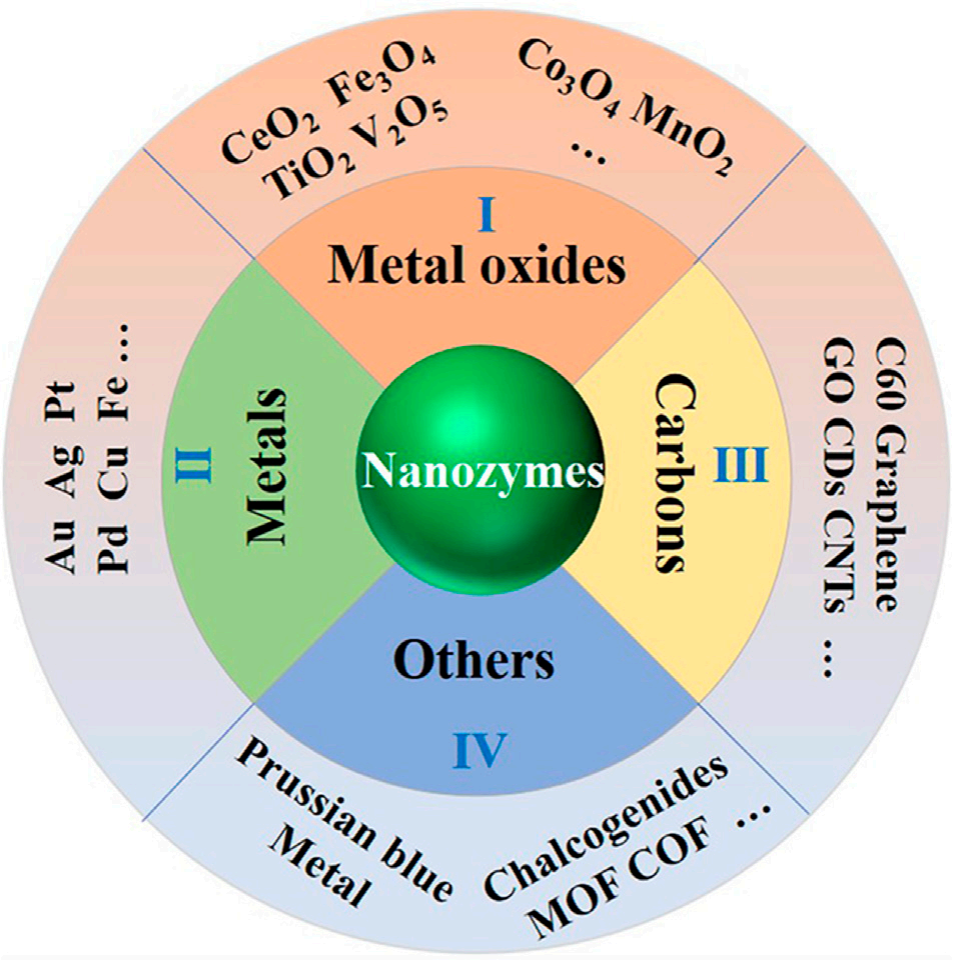
Schematic presentation of nanozymes classifications (metal-, metal oxide-, and carbon-based nanozymes and other nanozymes like MOF, COF, etc.).

Generally, nanozymes can be classified as four groups according to the components, that is, metallic-based, metal oxide-based, carbon-based, and other nanozymes (Figure 4). Metallic-based nanozymes include metal nanoparticles, such as gold nanoparticles (Au NP), platinum nanoparticles (Pt NP), and palladium nanoparticles (Pd NP). To enhance their catalase activity, some bimetallic nanozymes include Au@Pt, Pt@Pd, and Au@Ag that are developed (Nandhakumar et al., 2020). Even ternary metallic nanozymes like AgAuPt NP and PtPdAu NP were explored. Despite their advantages like being easy to be prepared and modified, metallic-based nanozymes may suffer from disadvantages including metal toxicity and spontaneous aggregations.

Unlike metal-based nanozymes, metal oxide-based ones possess abundant groups with different functions. For example, Fe_3_O_4_ nanozymes can be used as peroxidase-, catalase-, or oxidase-like enzyme, and CeO_2_ nanozymes can act as superoxide dismutase, catalase-, and oxidase-like enzymes (Nicolini et al., 2015). In addition, there are many other metal oxide-based nanozymes, such as CuO NP, MnO_2_ NP, V_2_O_5_ NP (Yang F. et al., 2018), and so on. With the aid of surface functional groups, metal oxide-based nanozymes are more stable and easier to be modified.

Carbon-based nanozymes mainly include carbon nanotubes (CNT), graphene oxide (GO), and carbon nanodots, which were known as carbon-based nanozymes (Garg and Bisht 2016). For example, Cui has developed a combined hydrothermal/hydrogen reduction method for mass production of spiral carbon nanotubes by pyrolysis of acetylene (Cui et al., 2011). Early in 2009, Wang et al. described an electrochemiluminescence (ECL) sensor based on CdS nanocrystals formed on the surface of multiwalled carbon nanotubes (CdS/MWCNT) (Wang et al., 2009). With the peroxidase-like activity of MWCNT, CdS/MWCNT can react with H_2_O_2_ to generate strong and stable ECL signals. Such examples can be found in many other applications like environmental engineering or even synthetic chemistry (Sun et al., 2018).

Recently, a variety of new nanomaterials have been found to mimic the activity of enzymes, such as metal–organic frameworks (MOFs), covalent organic frameworks COFs, and Prussian blue (PB) (Zhou et al., 2020). Because of its porosity and large specific surface area and the diversity of structures and functions, MOFs have been widely concerned and applied in a number of crucial sensing, energy, and catalysts domains, including toxins detection, gas storage and separation, and electrocatalysis (Lustig et al., 2017; Li et al., 2018). For example, Zhou et al. report the first chiral nanozyme based on mimicking a natural enzyme and the superior structure of COFs, which showed higher activity than the natural enzyme (Zhou et al., 2020). Li et al. developed a new kind of microelectrode for *in vivo* monitoring of H_2_O_2_ by electrodeposition of PB onto CNTs assembled carbon fiber microelectrodes (Li et al., 2016). All the methods are based on the excellent properties of the newly developed nanozymes.

### Functions and Performances

As a specific kind of artificial enzymes (Ⅱ), nanozymes behave desirable functions beyond catalysis. For example, from metal to metal oxides, then to carbon, a variety of sources can be provided to synthesize nanozymes, which make them readily available. Many nanozymes exhibit multienzymes functions by mimicking different kinds of natural enzymes. For example, depending on pH, CeO_2_ NP and Au NP can exhibit superoxide dismutase, peroxidase, and catalase activities, which is mainly dependent on their kinetic characterization (Wu et al., 2019c). Usually, Michaelis−Menten kinetics experiments are carried out to compare with those natural enzymes. By this means, the standards in terms of the substrate specificity (Km), catalytic rate constant (kcat), and catalytic efficiency (kcat/Km) can be united.

**FIGURE 5:**
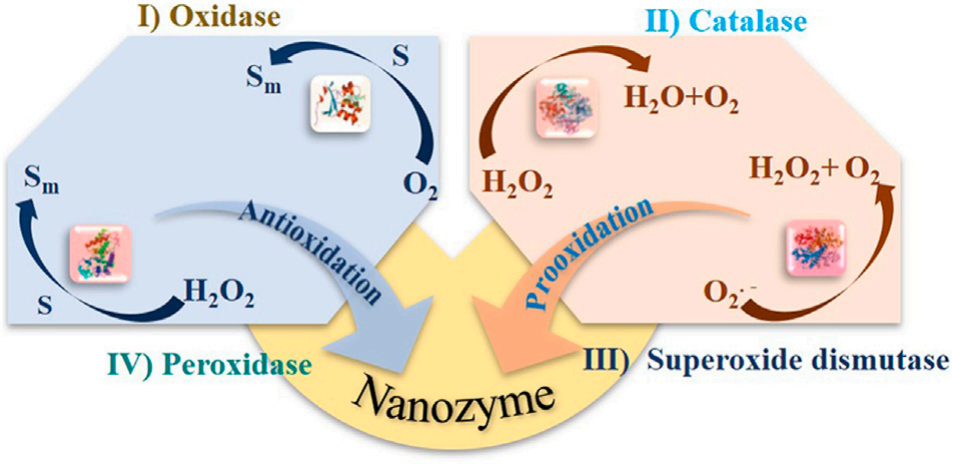
Functions and catalytic mechanisms of nanozymes (S, substrate).

In general, nanozymes have the oxidase-, hydrolase-, superoxide dismutase-, and catalase-mimicking activity (Figure 5). The catalytic mechanisms and kinetics have been widely discussed regarding conditions like pH, temperature, or even dissolved oxygen. However, as mentioned above, several different functions can be found in one type of nanomaterial, which are pH dependent or related to structural properties (size, morphology, surface groups, defects, etc.). Due to the complex interdependence between physicochemical properties and catalytic characteristics, a guide is needed to engineer and design nanozymes.

## Engineering and Modification

Many studies have revealed that the structural properties like size, morphology, and surface groups are vital contributors to the catalytic activity of nanozymes (Liu and Liu 2017; Jiao et al., 2019). Typically, the specific surface area of nanoparticles increases as their size decreases, which result in a serious shortage of coordination number of surface atoms. Therefore, the surface active sites increased, and the catalytic efficiency is enhanced. That is, the smaller the nanozymes are, the higher their catalytic activity is. In this regard, the enzyme activity can be adjusted by controlling the size.

Besides, during the reaction process, the morphology and interface structure of nanozymes may change with different reaction conditions and thus have effects on the catalytic performance (Vernekar et al., 2016). By selectively exposing the crystal surface with high activity or specific energy, the catalytic activity of nanoparticles can be improved a lot (Kuang et al., 2014). The morphology-dependent nanozymes can be ascribed to the different lattice arrangement of atoms with different appearance structures, which lead to different surface activity and catalytic performances.

**FIGURE 6:**
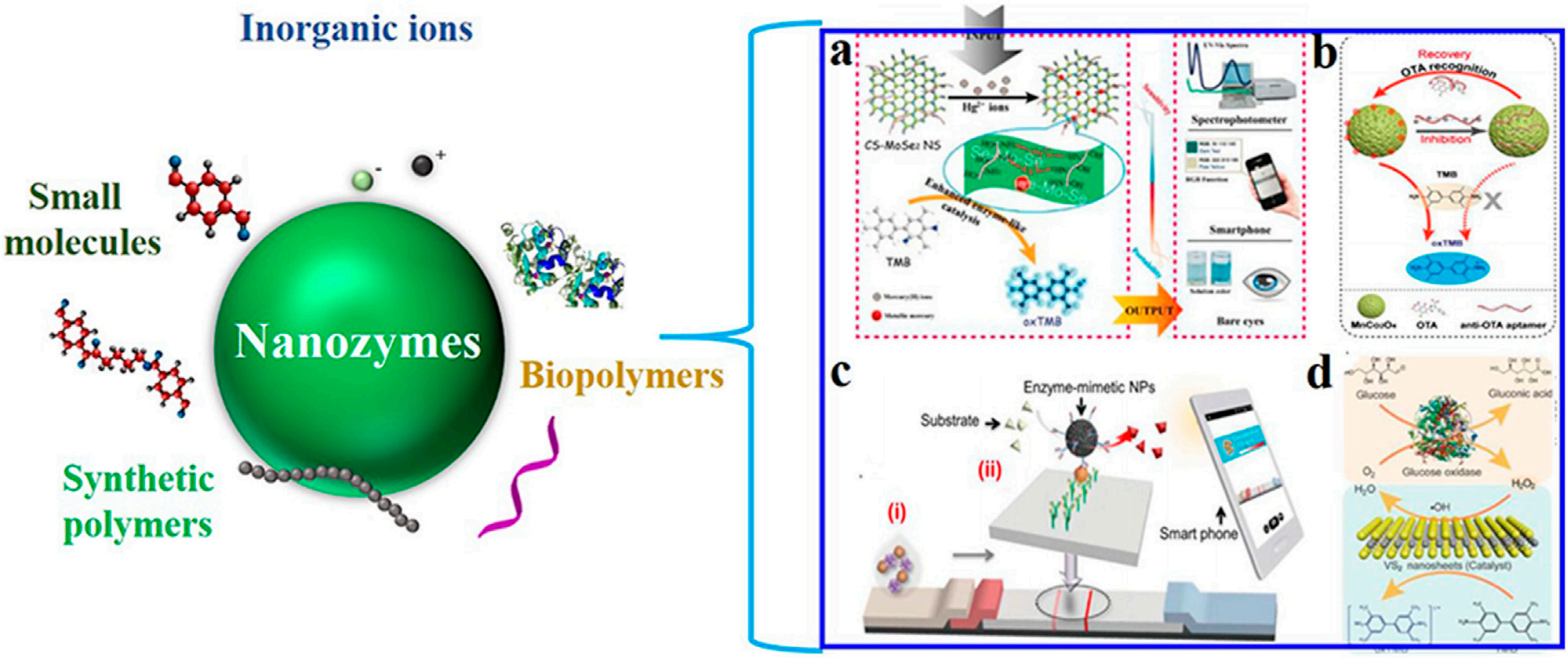
Modification of nanozymes and their application in constructing analytical techniques (**(a)**: Huang et al., 2019b; **(b)** Huang et al., 2018a; **(c)** Kim et al., 2015; **(d)** Huang et al., 2018b).

What is more, the functions of nanozymes can be reformed via a variety of surface modification means (charges, coatings, functionalization, and loadings). Based on this, both target recognition and target-dependent catalytic activities can be achieved by surface engineering strategies. Such successful modification examples include ions (Huang et al., 2019c), small molecules (Chang et al., 2016), nucleotides and nucleic acids (Huang et al., 2018a), amino acids and peptides (Fan et al., 2017), proteins (Su et al., 2019), and polymers (Wu Y. et al., 2019) (Figure 6). The operation can strengthen the functions of nanozymes and extend their applications. Specifically, those nanozymes with unique surface can realize sensitive and specific recognition and detection of analytes.

## Application in Food Contaminants Detection

Since food contaminants have posed great threat to human health and also show huge risks in food safety, it is essential to develop analytical methods for analyzing food contaminants for food safety supervision and risk assessment. Nevertheless, it remains great challenge to achieve rapid detection of food contaminants, and there are still some technical limitations to be solved. The development of cost-effective, rapid response, high sensitivity, and selectivity detection method for toxins has significant market prospects and huge social benefits. Taking advantage of the physiochemical properties of nanozymes, they are supposed be a potential candidate in improving the performance of analytical methods.

In this section, different classes of food contaminants are first introduced, including toxins, pesticide residues, food additives abuse, and microorganism, as well as their application and properties. Next, nanozymes-based analytical methods are carefully discussed to prove the good detection performance, especially for food contaminants with certain limit quantity. Immunoassays, a system based on biochemical recognition that can sensitively convert concentrations of analytes into signals, are introduced in food contaminants detection.

### Toxins

**FIGURE 7:**
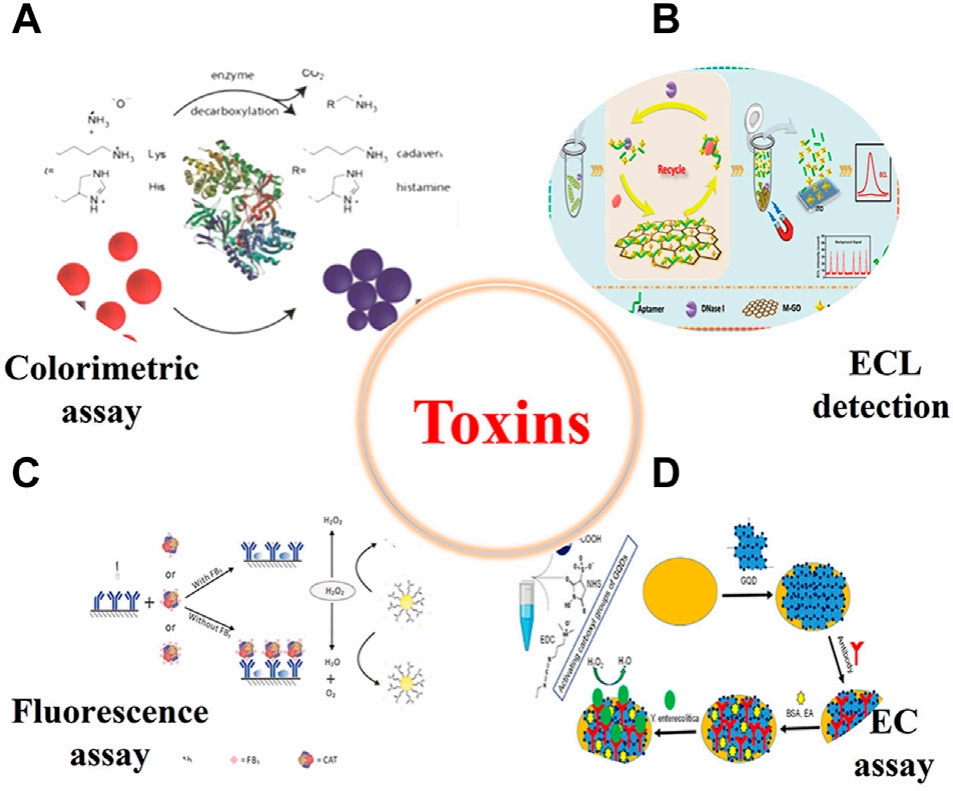
Representative illustration of nanozyme-based method for the detection of all kinds of toxins in food samples (EC, electrochemistry) (**(A)** Nilam et al., 2017; **(B)** Yang et al., 2021; **(C)** Lu et al., 2018; **(D)** Savas and Altintas 2019).

A toxin can be something produced by an organism that interferes with the action of other lives and cause poisoning in human body (Harms et al., 2018). Trace amount of the toxins in human body can cause biological damage, even resulting in death. For example, mycotoxins are secondary metabolites produced by some fungi (mainly *Aspergillus*, *Penicillium,* and *Fusarium*) during the growth, which can easily cause physiological abnormalities in humans and animals (Zain 2011). The mycotoxins can enter the food chain through contaminated grains or the products of animals (e.g., milk, meat, and eggs) that were fed with mycotoxin-contaminated feed. To ensure food safety and guarantee the human health, it is of great significance to develop powerful methods to monitoring the trace level of toxins in food samples (Figure 7).

#### Detection of Mycotoxins

The most common mycotoxins are aflatoxin, ochratoxin, fumonisin, zearalenone patulin, and deoxynivalenol. Due to their toxicological effects, the presence of mycotoxins in foods has severe implications on human and animal health even at very low concentration level (Cimbalo et al., 2020). Thus, it is essential to develop rapid methods for the detection of these mycotoxins in food products. For instance, based on CdTe/CdS/ZnS quantum dots, luminol, and HRP-modified gold nanorods, Wu et al. designed a ratiometric ECL aptasensor for AFB1 detection (Wu et al., 2017). The ratiometric aptasensors exhibited accurate and sensitive analytical performance for AFB1 with a good linear range from 5.0 pM to 10 nM with a LOD of 0.12 pM. The nanozymes-based immunosorbent assay has proved the superior performance of nanozymes in enhancing the detection sensitivity. Based on mesoporous SiO_2_/Au-Pt (m-SAP), an aptamer, and Fe_3_O_4_ magnetic nanoparticles (MNP), a nanozyme and aptamer-based immunosorbent assay (NAISA) was constructed for AFB1 detection (Wu et al., 2020a). In the work, m-SAP were prepared to act as signal labels, aptamer was adopted to recognize AFB1, and MNP facilitated to realize magnetic separation. The NAISA method showed a LOD of 5 pg ml^−1^, which is 600-fold lower than that of traditional ELISA method. Further, based on Au NPs-assisted triple cascade signal amplification, Hong et al. proposed a magnetic relaxing sensing method for the detection of AFB1 with a LOD of 0.453 pg ml^−1^ (Hong et al., 2021). Xu et al. reported an indirect competitive MOF-linked immunosorbent assay for AFB1, which overcome the low catalytic activity and poor stability of natural enzymes with 20-fold enhancement in sensitivity (Xu et al., 2021). Obviously, nanozyme-based detection techniques can greatly improve the sensitivity with specific structural properties and excellent catalytic activity.

#### Detection of Bacterial Toxins

Besides, the detection of bacterial toxins like *Escherichia coli* toxin, enterotoxins, and botulinum neurotoxins is discussed. For example, Ching et al. described the use of Au NP in a single lateral flow device for detection of botulinum neurotoxins A and B (Ching et al., 2012). If toxin is present, it binds with the gold-conjugated antibody and together flow to the test capture line, resulting in the resolution of a red line. This is a typical lateral flow immunoassay that can be extended to nanozymes-based analytical techniques. Shlyapnikov et al. reported a microarray-based immunoassay for the simultaneous detection of five bacterial toxins, including cholera toxin, *E. coli* heat-labile toxin, enterotoxins, and the toxic shock syndrome toxin (Shlyapnikov et al., 2012). The assay can be completed in less than 10 min with the LOD low to 0.1–1 pg ml^−1^ for water and to 1 pg ml^−1^ for food samples. Few nanozymes-based analytical techniques have been fabricated for the detection of bacterial toxins. However, nearly all the immunoassays involve the reaction of enzyme and H_2_O_2_, so it can be a creative way to apply nanozymes in the previously reported immunoassay by replacing the enzyme conjugated antibody.

#### Detection of Marine Toxins

Marine biotoxin is a kind of highly active special metabolic component in marine organisms, which can severely affect human health, economy, wildlife, and ultimately the ecosystem (Bano et al., 2020). By immobilizing BTX-2–bovine serum albumin conjugate on Au NP-decorated poly(amidoamine) dendrimers, Tang et al. developed an electrochemical assay for the fast screening of brevetoxin B (BTX-2) in food samples (Tang et al., 2011). The application of Au NP can improve the conductivity of dendrimers on the electrode. The BTX-2 assay was conducted with a competitive immunoassay using HRP-labeled *anti*-BTX antibodies and H_2_O_2_–*o*-phenylenediamine (OPD) reaction system. The method behaved a wide linear range of 0.03–8 ng ml^−1^ with a LOD of 0.01 ng ml^−1^. Based on a double-integrated mimic enzyme formed by Cu(OH)_2_ nanozyme and G-quadruplex/hemin DNAzyme, Liu et al. established an immunosensor for detection of microcystin-LR (Liu T. et al., 2019). In the strategy, Cu(OH)_2_ nanozyme acted as labels to capture the secondary antibody as well as a substrate for loading DNAzymes. The method had high activity for the ABTS chromogenic reaction, which realized the visual detection of microcystin-LR in the range from 0.007 to 75 μg L^−1^ with a LOD of 6 ng ml^−1^. Such double-integrated artificial enzyme showed a stable and catalytic ability to H_2_O_2_ and ABTS, further revealing the superiority of functional nanozymes.

#### Detection of Plant Toxins

Plant toxins, also known as phytotoxins, are secondary plant metabolites that have acute or chronic toxicity or pose antinutritional effects on people. The commonly detected that plant toxins include pyrrolizidine alkaloids, grayanotoxins, opium alkaloids, strychnine, ricinine, aconitine, aristolochic acid, and cardiac glycosides (e.g., digitoxin, digoxin). For example, Hu et al. proposed a sensitive colorimetric aptasensor for the quantitative detection of abrin using Au NP nanozyme (Hu J. et al., 2015). Au NP possesses the peroxidase-like activity that can catalyze TMB in the presence of H_2_O_2_ with color variations. The method behaved a linear range from 0.2 to 17.5 nM, with a LOD of 0.05 nM for abrin. Velmurugan et al. reported the fabrication of Co(OH)_2_-enfolded Cu_2_O nanocubes on reduced graphene oxide (rGO) to develop an electrochemical caffeine sensor (Velmurugan et al., 2016). The nanozymes had a good electrocatalytic activity towards the determination of caffeine in beverage samples. The sensor showed a linear range from 0.83 to 1,200 μM with a LOD of 0.4 μM. Furthermore, based on chitosan functionalized magnetic graphene oxide, Tang et al. developed an extraction method for efficient extraction and determination of alkaloids in hotpot (Tang et al., 2020). The study was carried out without using nanozymes. However, the detection was successfully conducted by the pretreatment of nanocomposites, which posed good guiding sense towards the nanozyme-based analytical techniques.

### Pesticide Residues

Pesticides are one of the major inputs used in agriculture to protect crops and seeds before and after harvesting (Bajwa and Sandhu 2014). Though they have contributed huge economic benefits to society, the pesticide residues left in the food materials can have deleterious effect on human health (Jiang et al., 2008). Moreover, widespread use of pesticides has caused serious concerns in food safety, because the residues are easily exposed to primary and derived agricultural products. Thus, in order to ensure food safety for consumers, many countries and organizations around the world have established maximum residue limits (MRL) for pesticides in foods (Jallow et al., 2017).

**FIGURE 8:**
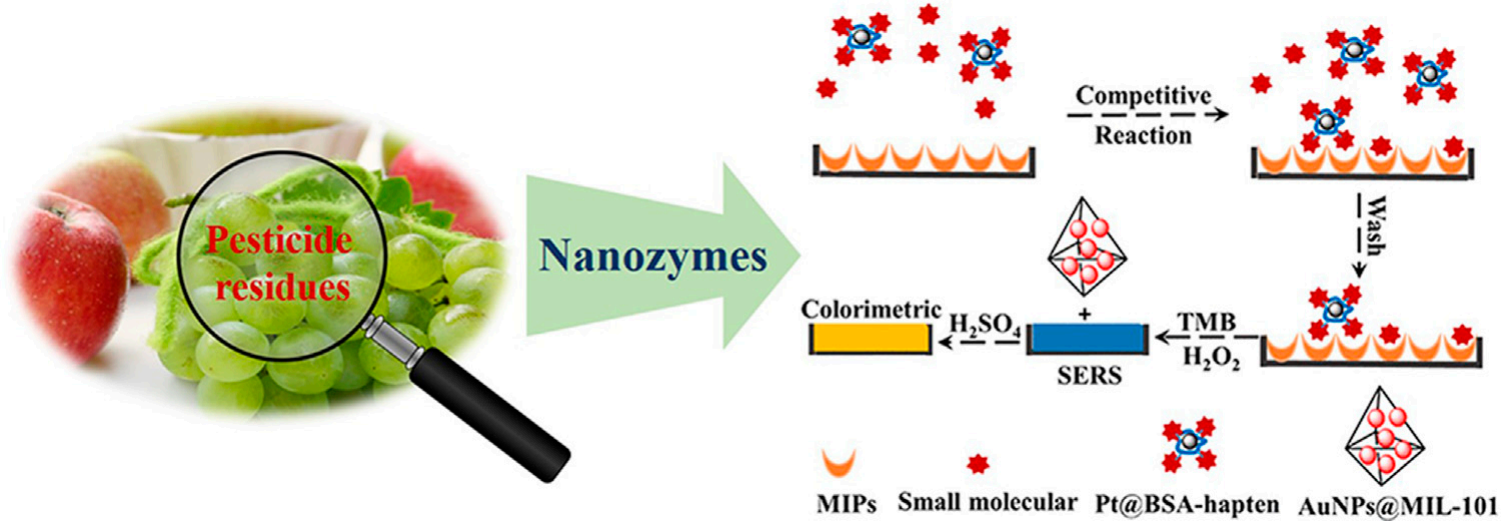
Representative illustration of nanozyme-based method for the detection of all kinds of pesticide residues in food samples (Yan et al., 2019).

On the other hand, due to the large amounts of pesticides currently being used, an increasing interest has been attracted for developing rapid screening systems to monitor their level in the food products (Liu W. et al., 2019). In this section, three kinds of pesticides include organophosphates (OPPs), neonicotinoids (NNOs), and triazines (TAs) that are introduced as analytes (Figure 8). To achieve robust detection of pesticides, several analytical techniques based on nanozymes are developed and highlighted.

#### Analysis of Organophosphates Pesticides

Among the toxic pesticides, organophosphorus pesticides (OPPs) have been reported as the major contaminants in the water, fruit, or medicinal plants (Yang Q. et al., 2018). For example, Wei et al. proposed a dual-mode strategy using nanoceria as nanozyme for methyl-paraoxon (MP) analysis (Wei et al., 2019). Based on the enzyme-like activity of nanoceria, MP could be hydrolyzed to *para*-nitrophenol (p-NP) with bright yellow color and characteristic absorption peak, which can be easily analyzed by the colorimetric and spectroscopic techniques. Both strategies showed LODs of 0.42 μM. To detect omethoate and dichlorvos and, at the same time, evaluate the activity of acetylcholinesterase (AChE), Huang et al. proposed a colorimetric paper sensor using γ-MnOOH nanowires (NWs) as nanozyme and 3,3′,5,5′-tetramethylbenzidine (TMB) as a chromogenic indicator (Huang et al., 2019a). The concentration of pesticides and AChE activity can be measured by the changes in absorbance at 652 nm or blue color of oxidized TMB products. The paper-based test had LODs of 0.1 mU mL^−1^ for AChE, 10 ng ml^−1^ for omethoate, and 3 ng ml^−1^ for dichlorvos.

#### Analysis of Neonicotinoid Pesticides

Neonicotinoid pesticide is a relatively new group of active ingredients with broad spectrum systemic action, low toxicity, and high insecticidal efficiency to mammals (Wu et al., 2020b). Weerathunge et al. reported a colorimetric assay for rapid detection of acetamiprid with acetamiprid-specific aptamer and Au NP nanozyme (Weerathunge et al., 2014). This approach can realize detection of 0.1 ppm acetamiprid within 10 min. Based on an aptamer against acetamiprid, multiple complementary strands (CSs), and gold nanoparticles (Au NP), a fluorometric assay was developed for the selective detection of acetamiprid (Bahreyni et al., 2018). The method can realize the detection of acetamiprid in a range of 5–50 nM with a LOD of 2.8 nM. In this work, apart from the nanozyme activity of Au NP, their quenching effect toward specific fluorescent materials was applied in analytical methods.

#### Analysis of Triazine and Other Pesticides

Once introduced into the crops, triazine pesticides can cause long-term negative effects due to their persistence. Another issue is their easy distribution into other parts of the environment, especially from soil into groundwater, one of the main sources of drinking water. Thus, it is a vital subject to develop analytical techniques of triazine pesticides by easy and cost-effective techniques in environmental chemistry.

Boruah and Das prepared Fe_3_O_4_-TiO_2_/reduced graphene oxide (Fe_3_O_4_-TiO_2_/rGO) nanocomposite with hydrogen peroxide activity and photocatalytic efficiency (Boruah and Das 2020). The colorimetric detection technique is applied for the sensing of atrazine using TMB as substrate molecules, which showed a LOD of 2.98 μg L^−1^ and a linear range of 2–20 μg L^−1^. Based on a competitive ELISA, Kwon et al. developed peroxidase-like mesoporous core-shell palladium@platinum (Pd@Pt) nanoparticle conjugated primary antibody as enzyme labels to detect atrazine (Kwon et al., 2020). The method leads to a high sensitivity with a LOD of 0.5 ng ml^−1^ and recoveries of 99–115%, demonstrating that atrazine and other herbicides and pesticides can be detected using this immunoassay. With the help of heteroatom-doped grapheme, Zhu et al. fabricated a colorimetric nanozyme sensor arrays for detection of the aromatic pesticides via the TMB/H_2_O_2_ system (Zhu et al., 2020a). Five different pesticides like fluroxypyr-meptyl, lactofen, diafenthiuron, bensulfuron-methyl, and fomesafen were successfully detected from 5 to 500 μM. Obviously, the inhibition effect of pesticides toward natural enzyme is also suitable for nanozyme, which can be effectively used to indicate the quantity of pesticides combining with TMB/H_2_O_2_ coloring system.

### Veterinary Drug Residues

**FIGURE 9:**
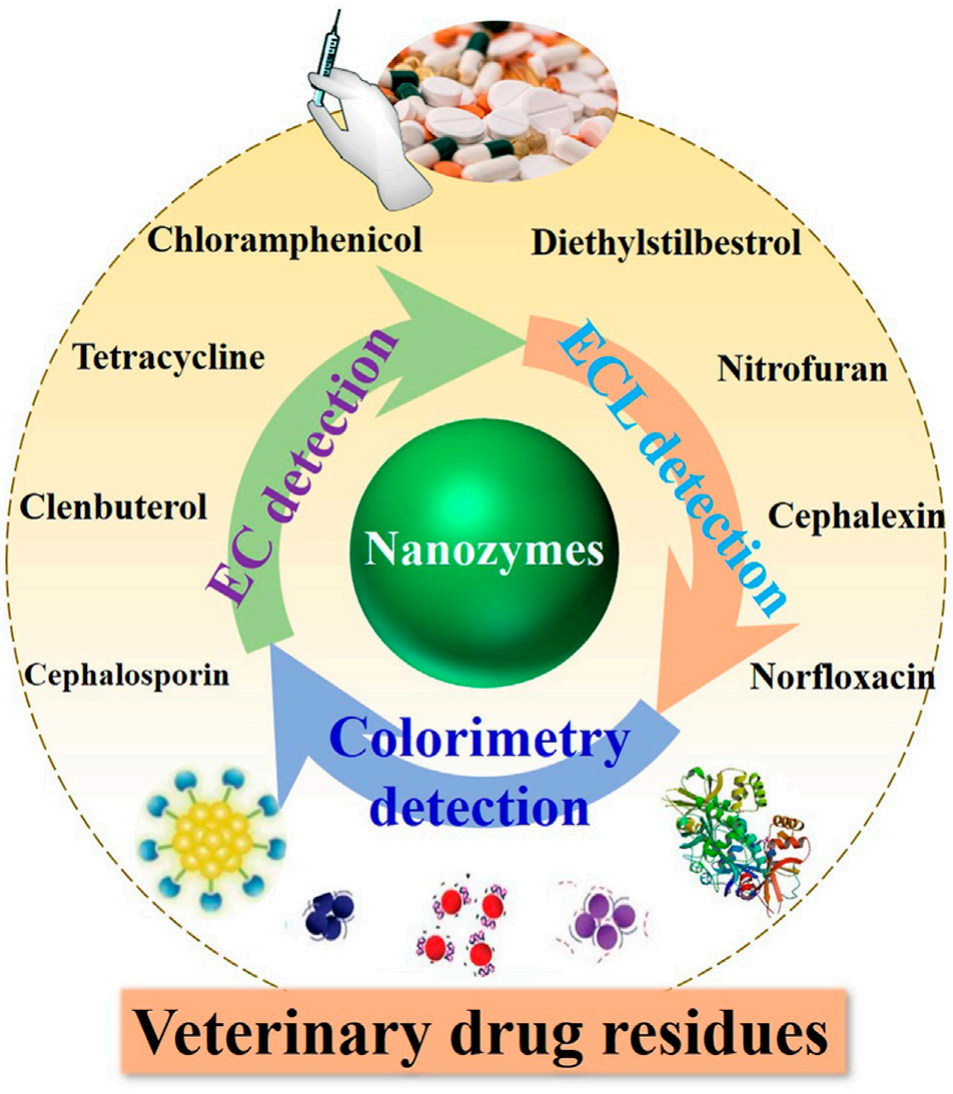
Schematic illustration of nanozymes in the design of detection platforms for veterinary drug residues as multifunctional sensing elements.

Veterinary drugs are a kind of substances, including pharmaceutical feed additives, which are often used to prevent, treat, and diagnose animal diseases or to purposely regulate animal physiological functions (Stolker and Brinkman 2005; Rocca et al., 2017). All veterinary drugs used in edible animals may cause residues in eggs, milk, and meat, which may contain parent compounds and metabolites or/and conjugates, and enter the human body via the food chain to produce direct toxic effects (Figure 9). Based on their functions, veterinary drugs can be classified as different groups such as antibiotics, anthelmintics, growth promoters, antiprotozoal drugs, trypanosomiasis drugs, sedatives, β-adrenergic receptor blockers, and so on (Winckler and Grafe 2001). The abuse of veterinary drugs can both cause direct harm to human health and the development of animal husbandry and the ecological environment (Masia et al., 2016). Therefore, it is critical to develop effective and rapid detection methods to screen food samples with veterinary drug residues.

#### Analysis of Antibiotics

Based on the peroxidase-like activity of gold nanoclusters (Au NC), Zhang et al. established a TMB/H_2_O_2_ colorimetric sensing method for tetracycline antibiotics (TCs) via TCs-specific aptamers (Apt) (Zhang et al., 2020). The sensor can accurately and reproducibly detect tetracycline in drugs and milk in the range from 1 to 16 μM with a LOD of 46 nM. In addition, Tian et al. established a ratio electrochemical biosensor for the quantitative detection of kanamycin based on signal amplification elements of planar VS_2_/AuNPs nanocomposites and CoFe_2_O_4_ nanozyme (Tian et al., 2020). The electrochemical aptasensor revealed a detection range from 1 pM to 1 μM with a LOD of 0.5 pM. Two main nanozyme-based analytical methods were introduced here. The first method is the colorimetric assay, which uses enzyme-mimic activity of nanozyme to produce color variations that can qualitatively determine analyte concentrations or adopt an instrument to quantitatively detect analytes. The other one is electrochemical assay, and nanozymes were used to catalyze TMB and generate oxidized species like oxidized TMB (oxTMB), behaving obvious characteristic signals for further analysis.

#### Analysis of Antibacterial

Antibacterial drugs are a class of drugs that can treat or prevent infectious animal diseases by inhibiting or killing the pathogenic bacteria. However, the abuse of such drugs can pose harmful effects on human health and the environment (Devasahayam, Scheld, and Hoffman 2010). Thus, various analytical methods have been developed for antibacterial drugs analysis. For instance, based on gold nanoclusters, Song et al. proposed a peroxidase-like activity enhancement assay for norfloxacin (Song et al., 2020). The linear relationship of norfloxacin monitoring was gained in the range of 1.25–8.0 μM with a LOD of 0.2 μM. In addition, He et al. constructed a biomimetic nano-enzyme-linked immunosorbent assay for sulfadiazine detection using Au@SiO_2_ nanoparticles labeling as markers (He J. et al., 2020). The method showed good stability with a LOD of 0.2 mg L^−1^ and recoveries from 78.00 to 90.96% in beef samples. Moreover, based on molecularly imprinted polymers and Cu(II) anchored unzipped covalent triazine framework, Ma et al. described an ECL assay for sulfa quinoxaline (SQX) using the luminol/H_2_O_2_ system (Ma et al., 2018). The method achieved good performance with a detection range of 1.0–20 pM and a LOD of 0.76 pM.

#### Analysis of Other Drugs

Other types of veterinary drugs like antiviral drugs and hormones are also easy to contaminate animal foods. Excessive use of these antiviral drugs will inevitably lead to drug residues in animals and eventually enter the human body via the food chain. Therefore, it is very meaningful to construct rapid method for detection of veterinary drug residues. For example, Ma et al. developed a colorimetric immunoassay for the detection of amantadine by introducing nanocube Pt as nanozyme labels (Ma et al., 2018). According to this protocol, antiviral drugs like amantadine can be detected with the sensitivity of 0.195 ng ml^−1^ for naked eyes and 0.134 ng ml^−1^ for optical detection. The proposed method not only outcompeted reported methods, but greatly improved the naked-eye and optical measurements as compared with conventional signal-off immunoassays.

### Pathogens

**FIGURE 10:**
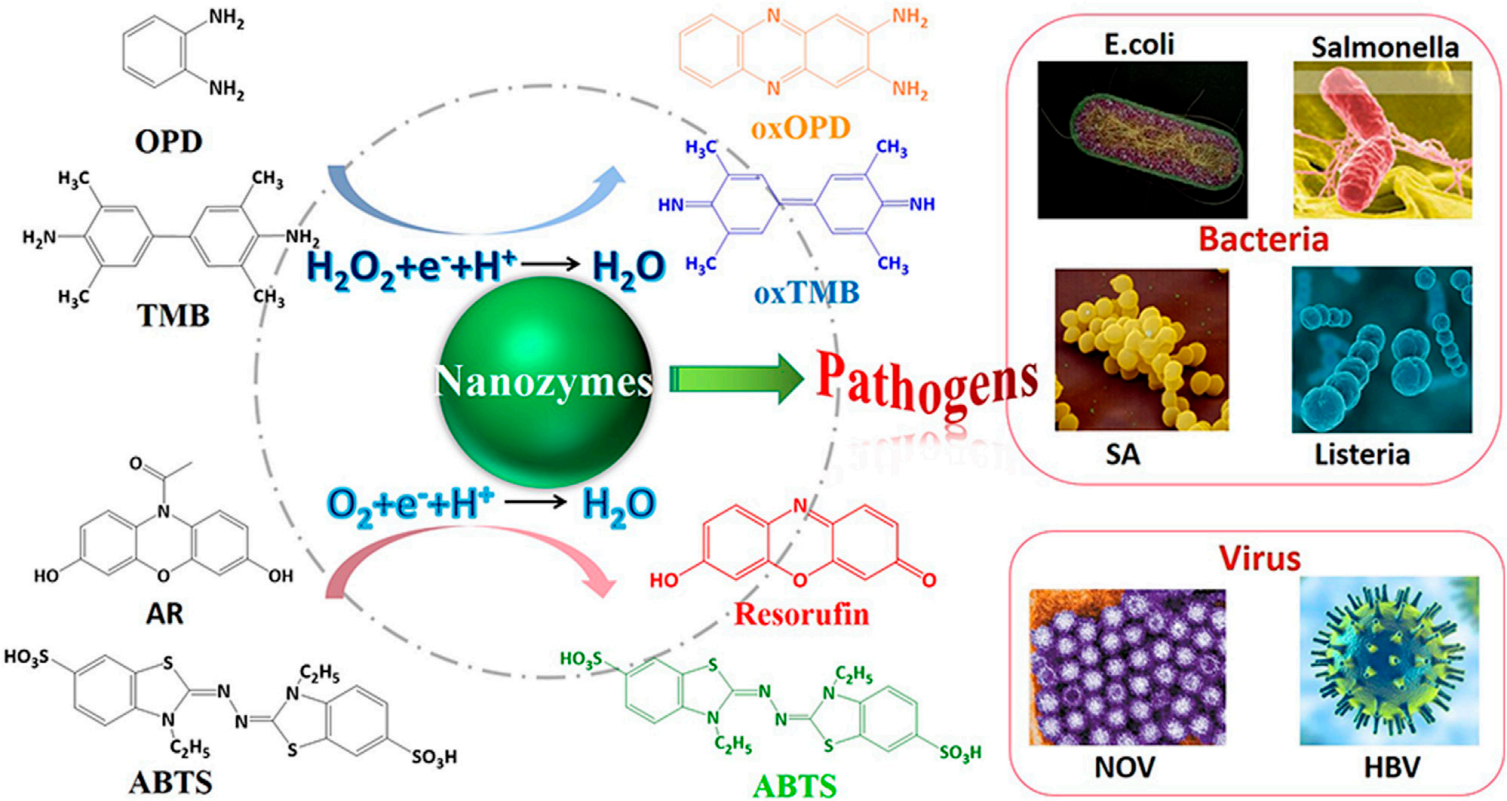
Schematic illustration of analytical techniques for foodborne pathogenic microorganisms based on nanozymes-based colorimetric assays.

Foodborne pathogenic microorganisms have attracted intensive attention in food safety, which can make bacteria in food multiply and even produce a large number of toxic metabolites. Poisoning accidents caused by mistakenly eating polluted foodstuff frequently occur. Traditional techniques suffered from limitation of low sensitivity, complex procedures, and time-consuming operations. Emerging analytical methods based on nanozymes have been widely developed, which make pathogens easier to be detected. In this section, various nanozyme-based assays for foodborne pathogens are described, involving colorimetric assay, lateral flow immunoassay, electrochemical assay, and so on (Figure 10).

#### Bacteria

Bacteria are the main categories of foodborne pathogens, including *E. coli*, *Salmonella*, *Listeria*, *S. aureus*, *Shigella*, *S. haemolyticus*, and *V. parahaemolyticus*. Among them, *E. coli O157:H7 (E. coli)* is a highly infectious pathogen that spreads widely in food and water and poses a major challenge to public health. Therefore, there is an urgent need to develop a new rapid method for detection of foodborne pathogens.

For example, Fu et al. proposed a two-step cascade signal amplification strategy for detection of *E. coli*, which combines *in situ* gold growth with nanozyme-catalyzed deposition and greatly improves the sensitivity of conventional gold lateral flow assay (Au NP-LFA) (Fu et al., 2020). The *in situ* engineering of nanozyme method achieved an ultrahigh LOD of 12.5 CFU ml^−1^, 400-fold enhancement compared with that of traditional Au NP-ICA. Han et al. prepared palladium-platinum (Pd-Pt) nanoparticles as enzyme probes to establish a sensitive LFA for detection of *E. coli* (Han et al., 2018). By using TMB substrate onto the test line, the assay exhibited an enhanced sensitivity of 9.0 × 10^2^ CFU ml^−1^ in milk, which was 111-fold higher than that of traditional Au NP-ICA. To get over the shackles of traditional LFA and build a flexible model, increasing studies have been carried out to develop a label-free and low-cost LFA, in which functional nanozymes are widely adopted to replace enzyme labeled antibodies and act as effective recognition agents to generate signals (Cheng et al., 2017; Liu et al., 2018; Wang H. et al., 2020; Liu, Wang, et al., 2020). According to previous reports, the colorimetric assay can become a versatile strategy for rapid detection of a wide variety of other bacteria and pathogens. Moreover, Zhang et al. designed a MOF@COF nanozyme to perform enhanced inhibition of bacteria like *E. coli* and *S. aureus*, which demonstrate the possibility of nanozymes using as potential antibacterial agents (Zhang et al., 2021). No matter in detections or antimicrobial applications, active centers, hierarchical nanocavities, and pore microenvironment within nanozymes always play important roles in their efficient catalytic activity.

#### Viruses

Apart from bacteria, viruses belong to foodborne pathogenic microorganisms that may contaminate the foods. However, a lack of knowledge about the viruses in food safety issues is reported. At present, the common foodborne viruses mainly include hepatitis A and hepatitis E viruses, rotavirus, astrovirus, enterovirus, and norovirus. Human norovirus (NoV) is one of the most common viruses that cause foodborne outbreaks worldwide (Patel et al., 2009). For example, based on the peroxidase activity of silver ion-incorporated Au NP (Au/Ag NP), Khoris et al. developed a colorimetric bioassay for detection of NoV (Khoris et al., 2019). Simply, NoV was captured by anti-NoV genogroup II antibodies and formed a sandwich structure with antibody modified Au NP. The *in situ* growth of Au/Ag was controlled by introducing Ag^+^/hydroquinone solution. When TMB/H_2_O_2_ was added to the wells, Ag ions were released from the surface of Au/Ag NPs and enhanced the oxidation of TMB with an intense blue color. The method showed a LOD of 13.2 c132 copies/g feces in the range of 10^2^∼10^6^ copies of viral RNA/mL. The strategy offers an alternative for practical deployment of the norovirus detection with simple pretreatment in contaminated food.

### Food Additives

Food additives are a kind of raw material added in food or in the process of food production, which aim to improve food edibility as well as the taste and aesthetic feeling of food. The excessive use of additives or illegal use of non–food additives will cause a series of food safety problems. High doses of food additives may have harmful long-term effects on animals such as cancer proliferation (Dolatabadi and Kashanian 2010). Therefore, the analysis of these unavoidable additives in food samples is important. In this section, nanozyme-based methods are discussed for the detection of food additives.

#### Analysis of Antioxidants

Antioxidants can prevent or delay food oxidation deterioration by reducing oxygen or free radical level around food and thus improve the stability and storage resistance. Many analytical procedures have been developed for the detection of antioxidants. In this part, nanozymes-based electrochemical and colorimetric assays are introduced to analyze antioxidants. For example, based on spiny Au-Pt nanotubes and horseradish peroxidase, Wu et al. proposed an electrochemical biosensor for the simultaneous determination of butylated hydroxyanisole (BHA) and propyl gallate (PG) (Wu et al., 2016). The method showed a wide linear range of 0.3–50 mg L^−1^ and 0.1–100 mg L^−1^ for BHA and PG with LODs of 0.046 mg L^−1^ and 0.024 mg L^−1^. Moreover, BHA and PG were demonstrated by a simple visual detection method, which involved Au-Pt nanotubes, a kind of nanozymes, as catalyst and TMB as indicator. The combination of visual and electrochemical detection can greatly enhance the detection accuracy. In addition, Cui et al. designed and prepared a novel porphyrin-based porous organic polymer, which was adopted in the TMB/H_2_O_2_ reaction system for the evaluation of antioxidants, like ascorbic acid (AA), gallic acid (GA), and tannic acid (TA) (Cui et al., 2018). The catalytic activity of nanozymes is also widely used in constructing electrochemical sensors. For instance, Yue et al. reported an electrochemical sensor for sensitive detection of TBHQ by integrating molecularly imprinted polymers (MIP), PdAu NP, and reduced graphene oxide (RGO) (Yue et al., 2019), wherein MIP realized the specific recognition with TBHQ, GRO accelerated electron transfer, and bimetallic PdAu NP acted as a promising catalyst.

#### Analysis of Food Colorants

Food colorants are dyes or pigments that impart color added to food or drink or any nonfood applications. Among them, synthetic ones like tartrazine, ponceau 4R, allura red, and sunset yellow are widely used in food industry. However, continuous intake of synthetic ones may do certain toxicity to human body. Experiments demonstrated that a high dosage of the dye like amaranth might increase the incidence of malignant tumors in rats (El-Wahab and Moram 2013). So, the synthetic pigments allowed in some countries are regulated with strictly limited dosage. Nanozymes are popular in detecting colorants using the electrochemical sensors.

For example, based on TiO_2_/electroreduced graphene oxide nanocomposites, Qin et al. described a voltammetric sensor for simultaneous detection of ponceau 4R and tartrazine (Qin et al., 2020). The method achieved the limit of detections (LODs) with 4.0 and 6.0 nM for ponceau 4R and tartrazine, respectively. Similarly, Li et al. reported TiO_2_/ErGO nanohybrids for the electrochemical detection of allura red with enhanced electrocatalytic activity and voltammetric response, and the LOD is 0.05 μM (Li et al., 2020). Besides, CuS with different morphologies was studied and then applied in tartrazine and sunset yellow detection by voltammetric techniques (Li et al., 2019). The CuS nanoflowers constructed electrode realized the detection limits of 12 nM for tartrazine and 6 nM for sunset yellow, revealing the unique electrocatalytic activities of CuS crystals.

#### Analysis of Other Food Additives

Other additives that are commonly detected include preservatives, sweetener, and flavor enhancers. For example, based on the peroxidase-like catalytic activity of nanozymes, Xi et al. designed copper/carbon hybrid as potential enzyme mimetics to generate ROS for antibacterial therapy (Xi et al., 2020). Based on ZnO NP/MWCNTs modified glassy carbon electrode, Balgobind et al. developed a differential pulse voltammetry (DPV) technique for aspartame detection (Balgobind et al., 2016). For the detection of preservatives, Rather et al. proposed an electrochemical detection of parabens by depositing polyaniline film (PANI) and Au NP on the glassy carbon electrode. The square wave voltammetric response of ethylparaben (EP) shows a wide linear range from 0.1 to 5.10 nM with a LOD of 0.1 nM. In addition, using gold nanoparticle decorated on a molybdenum disulfide/chitosan (Au@MoS_2_/Ch) as a conductive matrix, Devi et al. constructed an electrochemical immunosensor for the detection of monosodium glutamate, a kind of flavor enhancers (Devi et al., 2019). A linear detection range was perceived from 0.05 to 200 μM, with a LOD and limit of quantification (LOQ) of 0.03 and 0.1 µM, respectively.

### Heavy Metal Ions

Due to their potential threat to the public health, heavy metal ions (Hg^2+^, Pb^2+^, Cd^2+^) in food has been of increasing concerns (Wu J. et al., 2021). Long-term intake of these heavy metal ions, even with trace amount in food, will cause some severe diseases, such as cognitive deficits, kidney failure, cardiovascular, and neurological disorders (Zhang et al., 2019). In addition, Cu^2+^ is an essential element at the trace level in human body. For example, it can play a catalytic action in heme synthesis, but the intake of large quantities can be toxic. It is therefore essential to monitor heavy metals in the food or drinking water. Currently, nanozyme-based analytical method is one of the frontiers in the detection of toxic heavy metal ions. So, to achieve rapid, simple, and sensitive detection of those heavy metal ions, many detection methods coupled with nanozymes have been developed.

For example, Liu et al. prepared an Au/Ni-Fe LDH/rGO nanocomposite that both acts as enzyme mimics and surface-enhanced Raman scattering (SERS) substrate for the removal and detection of organic mercury (MeHg) (Liu et al., 2021). Based on the nanozyme material, MeHg can be degraded and removed as well as detected with a LOQ of 10 nM, which is significant in terms of the multiple applications of nanozymes. Huang et al. reported a new chitosan-functionalized molybdenum(IV) selenide nanosheets (CS-MoSe_2_ NS) for the colorimetric sensing of Hg^2+^ (Huang et al., 2019b). With the principle of Hg^2+^ activated CS-MoSe_2_ NS nanozyme activities and the indicator of TMB, Hg^2+^ ions could be quantitatively and selectively monitored with a LOD of 3.5 nM. The method is based on the surface modification of nanozymes, and the catalytic activity can be selectively triggered by specific target, which could be an example for designing other specific nanozymes. Based on Ag-CoFe_2_O_4_/reduced graphene oxide (rGO) nanocomposites, Guo et al. established a dual colorimetric and SERS detection assay for the sensitive detection of Hg^2+^ with a LOD of 0.67 nM (Guo et al., 2018). For the detection of Pb^2+^ ions, Xie et al. proposed Au@PtNP nanozyme as a colorimetric probe based on the surface leaching of Au@PtNP nanozyme (Xie et al., 2020). By using the TMB/H_2_O_2_ coloring system, a LOD of 3.0 nM with a linear range from 20 to 800 nM was achieved. Liu et al. presented a facile strategy for selective detection of Cu^2+^ by combining the peroxidase-like nanozyme activity of gold nanoclusters with amino acid ambidentate nature (Liu et al., 2017). The nanozyme probe showed a linear range of 1–100 nM and a LOD of 0.1 nM using the TMB/H_2_O_2_ system. Besides, Wen et al. developed a nanozyme–SERS system for detection of fluoride based on reduced MnCo_2_O_4_/Au nanotubes, which revealed the key roles of •OH and O_2_^•–^ radicals in the catalytic mechanism of nanozymes (Wen et al., 2020). The constructed methods are adopting the inhibition principle of heavy metal ions (Cu^2+^) or anion (F^−^) toward the catalytic activity of nanozymes.

Various nanozyme-based analytical methods have been developed to analyze food contaminants aiming at achieving good selectivity, high sensitivity, and stability. The nanozyme-based methods may overcome some disadvantages involving high cost of natural enzyme, time-consuming procedures, and complicated operations. However, it remains a big challenge to obtain controllable and stable nanozymes in the enhanced methods. To compare the performance of nanozyme-based methods in food safety detection, Table 1 listed the detection parameters such as nanozyme classification, analytes, linear range, and limit of detection (LOD), as well as examples of different nanozymes.

**TABLE 1:** Reported nanozyme-based methods in food safety detection.

Classification	Nanozyme	Analytes	Analytical method	Linear range	LOD	References
Metal-based nanozymes	Au NP	Patulin	SERS	0.5nM∼1 μM	0.085 nM	Zhu et al. (2020b)
	Pt NP	Histamine	BIA	0.90∼2,699.18 μM	1.15 μM	Wang et al. (2020b)
	Pd NP	Iodine ions	Colorimetry	0∼6.25 nM	0.19 nM	He et al. (2020b)
	Au@Pt	Aflatoxin B_1_	NAISA	0.032∼3,202.36 nM	0.016 nM	Wu et al. (2020c)
	Metal oxide-based nanozymes	Fe_3_O_4_ NP	Phenol	Colorimetry	1.67 μM∼1.2 mM	3.79 μM	Wu et al. (2020a)
		CeO_2_@MnO_2_ NP	Glucose	PEC sensor	0.1 μM∼0.3 mM	0.07 μM	Wang et al. (2020c)
		CuO NP	Ascorbic acid	Fluorometry	0.75∼7.5 μM/12.5∼125 μM	29.2 nM	He et al. (2020c)
MnO_2_ NP		Paraoxon	Electrochemistry	0.36∼72.68 μM	0.09 μM	Wu et al. (2021b)
		V_2_O_5_ NP	Dimethylamine	Chemiresistive sensor	─	0.11 mM	Mounasamy et al. (2018)
Carbon-based nanozymes		GQDs	*Y. enterocolitica*	Electrochemistry	1∼6.23 × 10^8^ cfu mL^−1^	5 cfu ml^−1^	Savas & Altintas (2019)
		CoO_*x*_H-GO	Cyanide ions	Colorimetry	100 nM∼100 μM	32 nM	Lien et al. (2018)
			MoS_2_/*f*-MWCNTs	Chloramphenicol	Electrochemistry	0.08∼1,392 μM	0.015 μM	Govindasamy et al. (2017)
	Other nanozymes	LMOF-241	Aflatoxin B_1_	Fluorometry	─	0.15 μM	Hu et al. (2015b)
Prussian blue		S*. typhimurium*	NLISA	6×10^3^∼10^6^ cfu mL^−1^	6 × 10^3^ cfu mL^−1^	Farka et al. (2018)
TAPB-DMTP-COF		Pb(II) ion	Electrochemistry	0.005∼2.0 μM	1.9 nM	Zhang et al. (2018)
		VS_2_	Glucose	Colorimetry	5∼250 μM	1.5 μM	Huang et al. (2018b)

SERS, surface enhanced Raman scattering; BIA, biomimetic immunoassay method; NAISA, nanozyme and aptamer-based immunosorbent assay; PEC sensor, photoelectrochemical sensor; GQDs, graphene quantum dots; MoS_2_/*f*-MWCNTs, molybdenum disulfide nanosheets coated on functionalized multiwalled carbon nanotubes; CoO_*x*_H-GO, cobalt hydroxide/oxide-modified graphene oxide; OPs, organophosphate pesticides; NLISA, nanozyme-linked immunosorbent assay.

## Perspectives and Challenges

Along with their remarkable properties, nanozymes-based analytical techniques have been booming. To drive the development of nanozyme research in food safety, it is essential to open a new avenue that can solve the limitations of the exited analytical methods. Fortunately, most of nanozymes are applied in constructing rapid detection methods like fluorescence, colorimetry, electrochemistry, and biosensors, which has provided some potential opportunities to meet the demands of analytical science. In this view, we summarized the nanozymes-based analytical methods for the rapid and sensitive detection of food contaminants. Though nanozymes can enhance analytical performance, they are new artificial enzymes that are full of challenges remained to be addressed.

1) We explore principles and mechanisms of nanozymes. Although a large number of papers have been reported on nanozymes, few experimental studies focused on the theoretical work and mechanism clarification. It is of great importance to explore the fundamental principles and mechanisms of nanozymes, which can facilitate to reveal the rule of structure-activity relationship and guide the precise design of nanozymes with desirable applications.
2) We develop uniform system and standards. Nanozymes are built up from the concept of enzyme; however, the properties differ a lot from natural enzymes. So, it is difficult to characterize the nanozyme performance in a traditional way. For example, the Michaelis–Menten mechanism is popular in discussing natural enzymes, but it is clear that natural enzymes catalyze a reaction through a homogeneous medium, which is different from nanozymes that occur in a heterogeneous mechanism on the surface of nanomaterials. Thus, uniform system and standards should be constructed to better characterize nanozyme performance.
3) We engineer controllable and functional nanozymes. Since size, morphology, and surface groups pose effects on nanozyme activity and functions, it is favorable to achieve nanozymes with high performance. How to controllably engineer nanozymes and extend their functions by surface modification is an important direction.
4) We evaluate high-performance nanozymes. In developing improved analytical techniques, various nanozymes are reported for signal production and amplification. However, when applied to the real applications, the catalytic activity of nanozymes is still relatively low. Compared to natural enzymes, the types of nanozymes are limited, and nanozymes can hardly catalyze one specific substrate. Hence, it is in great need to develop nanozymes with high catalytic activity, various enzymatic activity, and good substrate selectivity.
5) We integrate distinct techniques. It is encouraging that nanozyme-based detection techniques are narrowing the gap to practical-oriented food analytical methods. But it is almost impossible to achieve all the advances in a single detection technique. Thus, it is an alternative to develop nanozymes-based techniques with multimodes for the rapid, accurate, sensitive, and selective detection of food contaminants. For instance, it can greatly improve the specificity and selectivity of nanozymes by coupling with molecular imprinting technique.

In general, nanozymes are in the early stages of the development of the second generation artificial enzymes. The powerful functions of nanozymes make them popular from *in vitro* detection to *in vivo* monitoring, and we believe that they will have great potential in the analysis of food contaminants in the near future. The above challenges will be the next frontier for further nanozyme research.
